# Unveiling the Phytochemical Profile and Anti‐Cancer Potential of *Lantana camara* Leaf and Root Extracts Against MCF‐7, HepG2, and A549 Cancer Cell Lines

**DOI:** 10.1002/fsn3.70915

**Published:** 2025-09-11

**Authors:** Somia Shehzadi, Sana Noreen, Hassan Imran, Ali Ikram, Muhammad Tayyab Arshad, Kodjo Théodore Gnedeka

**Affiliations:** ^1^ University Institute of Medical Laboratory Technology The University of Lahore Lahore Pakistan; ^2^ University Institute of Diet and Nutritional Sciences The University of Lahore Lahore Pakistan; ^3^ University Institute of Food Science and Technology The University of Lahore Lahore Pakistan; ^4^ Functional Food and Nutrition Program, Faculty of Agro‐Industry Prince of Songkla University Hat Yai Songkhla Thailand; ^5^ Togo Laboratory: Applied Agricultural Economics Research Team (ERE2A) University of Lomé Lome Togo

**Keywords:** Annexin‐V, apoptosis, cytotoxicity, liver cancer, VEGF

## Abstract

Cancer is a life‐threatening disease; liver cancer, breast cancer, and lung cancer account for high prevalence worldwide. The *Lantana camara* (LC) plant is known for its cytotoxicity in treating certain diseases. However, the effect of leaf and root extract of LC on induction of apoptosis and lowering the proliferation of cancerous cells was estimated in the current study. Leaf and root extracts of LC were prepared using the rotary method, in which mineral analysis was done quantitatively through the spectrophotometry technique. In contrast, phytochemical composition was assessed both quantitatively and qualitatively. Fifteen bioactive compounds were detected from the LC root extract. The major compounds found were lupeol (52.94%), Lup‐20(29)‐en‐3‐one (8.231%), 9‐octadecynoic acid (21%), N‐hexadecenoic acid (16%), Phytol (5.842%), Hexadecenoic acid (5.301%), Caryophyllene oxide (4.772%) and 2,3‐Dihydro‐2,5‐dihydroxy‐6‐methyl‐4H‐pyran‐4‐one (3.018%). Cytotoxicity was estimated through the MTT assay by using two different sourced cell lines as control (HUVECs and Vero). VEGF was done to confirm the proliferation of treated cells; apoptosis was examined by Annexin‐V and Hoechst 33342 staining; cell viability was checked through the trypan blue and crystal violet staining method. Phytochemical analysis and antioxidant activities showed better results in the LCroot extract than the LCleaf extract. Similarly, DPPH measured for antioxidant analysis also confirmed the better results in LCroot (178.921 ± 0.12) (*p* < 0.01). In cell culture experiments, LCroot extract showed high cytotoxicity and morphological changes in MCF‐7, HepG2, and A549 cancerous cell lines, but a reduced cytotoxic effect was observed in non‐cancerous cell lines (HUVECs and Vero). ELISA for apoptosis and angiogenesis confirmed the significantly increased apoptosis (*p* < 0.0001) (Annexin‐V) and reduced angiogenesis/proliferation (VEGF). Further, the Hoechst 33342 staining method also confirmed the increased apoptosis in treated cancerous cells with 50 μg/mL LCroot extract as compared to untreated groups and normal HUVECs.

## Graphical abstract

In cell culture experiments, LC‐Root extract showed high cytotoxicity and morphological changes in MCF‐7, HepG2, and A549 cancerous cell lines, but no effect was observed in non‐cancerous cell lines (Vero). ELISA for apoptosis and angiogenesis confirmed the significantly increased apoptosis (*p* < 0.0001) (Annexin‐V) and reduced angiogenesis/proliferation (VEGF). This study demonstrated the efficacy of the LC‐Leaf and Root extracts in inducing apoptosis and cytotoxic effects on cancerous cells, but they have no harmful effects on normal cells.

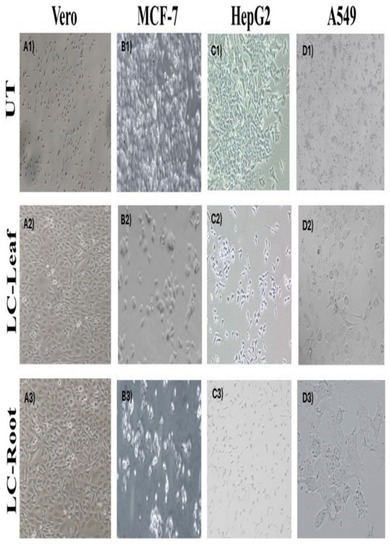

## 1 Introduction

Cancer is a condition which is characterized by uncontrolled cell division. Cancer growth is caused by a higher proportion of proliferating cells rather than a faster rate of cell division (Taha et al. 2023). The significant morbidity and mortality rate of hepatocellular carcinoma (HCC) makes it a global health concern. The third leading cause of cancer‐related death worldwide is HCC. Prolonged infection with the hepatitis B virus (HBV) and hepatitis C virus (HCV) is a significant risk factor for the development of HCC, which is also caused by metabolic disorders and excessive alcohol consumption (Sun et al. 2020). Breast cancer (BC) is the most common type of cancer in women. It is estimated that every year, about 2.3 million new cases of BC are discovered worldwide (Łukasiewicz et al. 2021; Tamanna et al. 2024). Early detection is crucial for hormone therapy, radiotherapy, and surgery, but there is a high risk of recurrence or metastatic cancers. Lung cancer is the most prevalent cancer in the world in terms of both diagnosis and cancer‐related fatalities (Lin et al. 2016; Chaurasia et al. 2023). According to GLOBOCAN 2020, there were approximately 1.8 million lung cancer deaths (18.0%) and 2.2 million new cases of lung cancer (11.4%) in 2020 (Cao et al. 2024). Different risk factors, such as the prevalence of smoking, environmental pollution, and even dietary habits, contribute to the variation in incidence among countries (Jemal et al. 2018). Vascular endothelial growth factor (VEGF) is a well‐defined marker for angiogenesis or proliferation, secreted by both stromal and tumor cells, such as macrophages, endothelial cells, and fibroblasts. VEGF has several functions in the tumor microenvironment, including angiogenesis's primary stimulus, endothelial cell sprouting, and increased vascular permeability (de Abreu et al. 2024; Schlüter et al. 2018). The urgent need for cancer treatments without damaging healthy cells has led to the identification of botanical compounds for cancer treatment due to their low side effects (Hu et al. 2017).

As new compounds with selective cytotoxic activity and fewer adverse effects are needed to address the noxious health issues associated with existing anti‐cancer drugs (Ikram et al. 2025; Negi et al. 2019), therefore, plants serve as valuable sources of bioactive compounds as they possess an innate ability to synthesize therapeutic compounds, aiding in the preparation of new drugs with high therapeutic value for various diseases (Omara et al. 2020). Based on fossil records, the earliest known human usage of plants as medicines dates to the Middle Paleolithic Period, around 60,000 years ago (Ntalo et al. 2022). LC, a natural botanical, has been used for centuries in medicine. LC is a neotropical shrub with a triangular stem and a strong aroma. It grows to 1–3 m in length and can be broadened to 2.5 m. Leaves are egg‐shaped, ovate rectangles, and green, enclosed by jagged hairs. Flowers are small, orange, and vary in color as they mature. The plant has a tiny calyx, lean limbs, stem, and ovules. Roots are well‐built and provide new shoots even after constant cuttings (Zhou, Kuang, et al. 2024; Kato‐Noguchi and Kurniadie 2021). LC, a plant rich in essential minerals like calcium, magnesium, potassium, iron, and zinc, has significant antioxidant activity due to its flavonoids, phenolic compounds, and terpenoids, which help scavenge free radicals, reducing oxidative stress (Ntalo et al. 2022; Sahu et al. 2022). LC is a versatile plant used globally for treating various diseases, including tumors, cancers, stomach aches, fevers, influenza, measles, chicken pox, sores, cold fevers, rheumatisms, high blood pressure, asthma, bronchitis, and stomach aches. Its leaves and flowers produce tea, which treats stomach aches, fevers, and influenza, while its crushed root prevents stomach aches (Shah et al. 2020).

The study aimed to estimate the bioactive composition, mineral content, and antioxidant property of LC‐Leaf and Root and evaluate the anti‐cancer activity of LC‐Leaf and LC‐Root extracts on liver, breast, and lung cancer cell lines compared with non‐cancerous cells.

## 2 Materials and Methods

### 2.1 Collection of LC and Extraction of LC‐Leaf and LC‐Root

The LC leaves and roots (scientific name: *Lantana camara* Linn.; family: Verbenaceae; Flora of Pakistan Plant number: 135) were gathered from Daman‐e‐Koh Islamabad, Pakistan, and were authenticated by an expert in the Department of Botany, The University of Lahore, Lahore, Pakistan. For extraction, LC‐leaves and LC‐roots were cleaned with distilled water (dH_2_O) and dried at 30°C. The plant materials were then ground into a fine powder using a grinding machine and stored in an airtight container in preparation for extraction. The 2 kg of powdered LC‐leaves and LC‐roots were immersed in ethanol and vortexed for 5 min. The mixture was then filtered through Whatman No. 1 filter paper. The filtrates were placed in a vacuum rotary evaporator set to reduced pressure and allowed to evaporate at 37°C. The resulting crude extracts were then kept for later use at 4°C.

All chemicals for phytochemical analysis were purchased from Merck, Germany. Chemicals for cell line experiments were purchased from Thermo Fisher Scientific, United States and cell lines were obtained from the cell culture laboratory at The University of Lahore, Pakistan.

### 2.2 Proximate Analysis

To ascertain the moisture and crude protein content of the LC leaves and roots, proximate analysis was performed as stated by AOAC (2005) (Produtos Agroindustriais 2013).

### 2.3 Mineral Analysis

An atomic absorption spectrophotometer (AAS) (Unicam Model 929, England) was used to determine iron, zinc, calcium, magnesium, and manganese; a flame photometer (Cole‐Parmer, EW‐83055‐02) was used to determine sodium and potassium. Spectrophotometry was used to determine phosphorus, as previously mentioned (Noreen et al. 2023).

### 2.4 Extraction Procedure

Freshly picked leaves and roots were allowed to air dry before being ground into powder using a mechanical grinder. 150 mL of ethanol was then used to apply samples for extraction in a Soxhlet apparatus. Subsequently, the extracts were concentrated to dry mass in a fume hood at room temperature and filtered through a Whatman No. 1 paper filter. The yield of the extract was assessed, and any leftover material was retained in the dark for later use. Samples were assigned to the designations LC‐Leaf (leaf extract) and LC‐Root (root extract). The dry extracts were dissolved in distilled water (dH_2_O) and ethanol to determine the final concentrations.

### 2.5 Quantitative Analysis

#### 2.5.1 Determination of Total Phenolic Content

Using the spectrophotometric approach, the Total Phenolic content (TPC) of extracts was assessed. To make the ethanolic solution, 500 μL (1 mg/mL) of extract, 10% Folin–Ciocalteu's reagent, 2.5 mL of 7.5% Na_2_CO_3_ and 2.5 mL of water were mixed. The mixture was then stirred to homogenize the dilution. Similarly, the blank was concurrently made with 0.5 mL of ethanol instead of extract. Samples were incubated in a thermostat at 45°C for 45 min in the dark. A spectrophotometer was used to detect absorbance at wavelength of 730 nm. To obtain the sample mean values, a triplicate setup was maintained. Gallic acid calibration curves were created in the 20–100 μg/mL range. Finally, the content of phenolics was reported in terms of gallic acid equivalents (mg GAE/g dry weight (d.w)) (Akuru et al. 2022).

#### 2.5.2 Determination of Total Flavonoids

The AlCl_3_ colorimetry method determined LC extracts' total flavonoid content (TFC) by following the previously reported method (Molole et al. 2022).

### 2.6 Detection of Alkaloids

The industry standard procedure was used to estimate the two extracts' total alkaloid content (TAC) (LC‐Leaf & LC‐Root). For this purpose, 2.50 g of each extract was taken in the beaker with 200 mL of 10% CH_3_COOH in ethanol, and they were left to incubate for 4 h at room temperature. Next, a dropwise addition of concentrated NH_4_OH was made until total precipitation occurred. The supernatant was then discarded, and the residues were cleaned using 20 mL of 0.1 M NH_4_OH. Finally, the filtrate residue was oven‐dried, and the proportion of alkaloids was determined (Kancherla et al. 2019).

$$\text{Percentage Alkaloid}=\frac{\text{Weight of Alkaloid}}{\text{Weight of Sample}}\times 100$$

### 2.7 Detection of Tannins

A gelatine test was utilized to determine the tannin content of the samples. The extract was mixed with 1% gelatine solution and sodium chloride (NaCl) to do this. White precipitate formation suggested the presence of tannins (Petchidurai et al. 2023).

### 2.8 Detection of Triterpenoids

To detect the presence of triterpenoids, 5 mL of each extract was mixed with chloroform (2 mL) and 3 mL of concentrated sulfuric acid. The appearance of a reddish‐brown color at the interface confirmed the presence of a triterpenoid (Das et al. 2014).

### 2.9 Detection of Saponins

The saponin content of the two extracts (LC‐Leaf & LC‐Root) was determined using the froth test. The reagent used in the test was 10 mL of distilled water. After giving the crude, dry extracts a good shake with 2 mL dH_2_O, they were left to stand for 10 min. The samples were thought to be positive for saponins if the height of the honeycomb froth remained at least 2 cm above the liquid's surface for 10 min. The presence of saponins was suggested by the appearance of stable foam (Kupnik et al. 2023).

### 2.10 Detection of Coumarins

To detect the presence of coumarins, the LC‐Leaf and LC‐Root extracts were mixed with 3 mL 10% NaOH. The yellow color in the solution confirms the presence of coumarins (Oduola‐Lawal et al. 2021).

### 2.11 Antioxidant Activities

#### 2.11.1 Ferric‐Reducing Antioxidant Power (FRAP) Assay

The total antioxidant capacity of the extracts was determined using the ferric reducing/antioxidant power (FRAP) using a slightly modified version of Benzie and Strain's methodology (Benzie and Strain 1996).

#### 2.11.2 ABTS Scavenging Assay

Radical cation decolorization is the basis for the 2,2′‐azino‐bis‐3‐ethylbenzotiazolin‐6‐sulfonic acid (ABTS) scavenging assay. According to Ben Nejma et al. (2017), the reduction of ABTS^+^ radicals depends on plant extracts' electron‐donating capability, which causes the radical to become decolored. 7 mM ABTS and 2.45 mMK_2_S_2_O_8_ were combined and incubated for 24 h, after which 300 μL of plant extract was added, and absorbance was determined at 745 nm. Whereas the inhibition percentage was assessed by using the formula (Ghagane et al. 2017):

$$\mathrm{SE}\%=\frac{\mathrm{Abs}.\;\text{of Control}-\mathrm{Abs}.\;\text{of Sample}}{\mathrm{Abs}.\;\text{of Control}}\times 100$$

where, SE: scavenging effect; Abs: absorbance.

#### 2.11.3 Iron (Fe^**2**+^)‐Chelating Analysis

The Barros et al. (2017) approach was slightly modified to calculate the extract binding ability to Fe^2+^. The ferrous ion was detected by producing a red ferrous ion‐ferrozine complex at a 562 nm wavelength. A combined mixture of 2 mM FeSO_4_, 40 μg/mL iron chelators, and 0.2 mM ferrozine was prepared. Absorbance was measured at 562 nm wavelength (Barros et al. 2017).

#### 2.11.4 DPPH Radical Scavenging Activity Assay

The 2,2‐Diphenyl‐1‐picrylhydrazyl (DPPH) free radical scavenging assay was performed as previously described by Zhou, Zhang, et al. (2024) with some slight modifications to assess the antioxidant potential of the LC‐Leaf and Root extracts. Fifty μL of the LC extracts were separately mixed with 5 mL of DPPH 0.005%. The mixture was dark for 30 min at room temperature, and ascorbic acid was used as a positive control. The wavelength of 517 nm was measured using a spectrophotometer (Zhou, Zhang, et al. 2024). The following formula was used to determine the DPPH scavenging effect:

$$\text{DPPH}\;\mathrm{SE}\%=\frac{\mathrm{Abs}.\;\text{of Negative Control}-\mathrm{Abs}.\;\text{of Extract}}{\mathrm{Abs}.\;\text{of Negative Control}}\times 100$$

### 2.12 Compounds Identification Through Gas Chromatography Mass Spectrometry (GC–MS)

*L. camara* extract compounds were identified by using Agilent Technologies. One milliliter per minute of helium gas was used. The split‐less column was filled with precisely 1 μL of the sample. At a rate of 10°C/min, the GC oven's temperature was raised from 60°C to 325°C and maintained at 280°C for 10 min. The temperature of the detector was 280°C, and the injector was 250°C. Each component's percentage composition was determined by integrating the GC peak area normalization. With a mass range of 15–500 m/z and an ionization voltage (EI) of 70 eV, these MS characteristics were analyzed. The NIST MS2011 library was used to characterize the analytical profiles based on their mass spectrum data.

### 2.13 Morphological Assessment of Cell Lines

In 24‐well plates with 10^5^ cells per well, Vero, MCF‐7, HepG2, and A549 cell lines were planted to examine the impact of LC‐Leaf and root extracts on cell shape at a concentration of 50 μg/mL. The cells were treated with LC‐Leaf and root extract at a concentration of 50 μg/mL for 48 h following a 24‐h incubation period. Next, photos were taken, and each group of cells was analyzed under an inverted microscope (OLYMPUS IX70‐S8F, Olympus Optical Co. Ltd., Japan) (Zhou et al. 2018).

### 2.14 Cytotoxicity Analysis via MTT Assay

The cytotoxicity of LC‐Leaf and Root extracts was evaluated on malignant (MCF‐7, HepG2, and A549) and two different non‐cancerous cell lines: Vero and Human umbilical vein endothelial cells (HUVECs) using the MTT (3‐(4,5‐dime thylthiazol‐2‐yl)‐2,5‐diphenyltetrazolium bromide) test. Cisplatin (20 μM) was used in all experiments serving as a vehicle control. The University of Lahore's cell culture facility provided all the cell lines. The production of purple‐colored formazan was measured to examine cytotoxicity. For 2 h, every 10^4^ cell lines were treated in a 60 μL MTT solution combined with cell culture Dulbecco Modified Eagle Medium (Invitrogen Inc., USA). The formazan was dissolved after adding sodium dodecyl sulfate (SDS) from Invitrogen Inc. USA, and the absorbance at 570 nm was determined (Shehzadi et al. 2024).

### 2.15 Enzyme‐Linked Immune Sorbent Assay‐ELISA

Sandwich ELISA was used to measure the levels of apoptosis (Annexin‐V) and angiogenesis (VEGF) in the treatment groups. In short, monoclonal antibodies against VEGF and Annexin V were applied to the microplate and then kept overnight at 4°C. Following three rinses, the coated plate was incubated for 16 h with 100 mL of the medium added. After adding 10% BSA (bovine serum albumin) to block, it was allowed to sit for 60 min. After blocking, a 1:200 horseradish peroxidase‐conjugated anti‐rabbit secondary antibody was applied and left at 4°C for 60 min. A chromogenic substrate (3,3',5,5'‐tetramethylbenzidine, Invitrogen) was added to stop the reaction after three washing cycles. To measure the optical density (OD) at 450 nm, a Molecular Devices microplate reader was employed.

### 2.16 Hoechst 33342 Staining to Detect Apoptosis in Cancerous and Non‐Cancerous Cells

The Hoechst 33342 dye was also used to identify the apoptosis of human cell lines, both healthy (HUVECs) and cancerous (MCF‐7, HepG2 and A549). All cells were treated with LC‐Leaf extract and LC‐Root extract (25 and 50 μg/mL) after being seeded on coverslips in a 6‐well plate. Following 48 h, the adhered cells were rinsed with PBS, fixed for 30 min in freshly made 4% paraformaldehyde (PFA), rinsed three times with PBS, and incubated for 5 min in Hoechst 33342 staining solution. Following treatment, cells were once more rinsed with PBS and treated with Antifade Mounting Medium (AMM). A fluorescent microscope was then used to detect apoptosis with condensed and shattered nuclei.

### 2.17 Crystal Violet Staining

Following treatment with the LC‐Leaf and Root extracts, 25 and 50 μg/mL each for 24 h, the final number of viable cells was calculated using the crystal violet assay following Kalampounias et al. (2024). Briefly, after the media from each experimental group was removed, the 96‐well plate utilized in this procedure was cleaned with PBS. The wells were filled with 2% ethanol and 0.1% crystal violet dye after cleaning. The incubation period was 15 min at room temperature. Disposing of the dye prevented cells from rising to the surface. The absorbance at 590 nm was then measured on a microtiter plate after the dye was dissolved by adding 200 μL of 1% SDS to each well (Kalampounias et al. 2024).

### 2.18 Trypan Blue Assay

Trypan blue was used as a dead cell detection agent to determine the percentage of dead cells. Following a PBS wash, the cells from the various experimental groups, Vero, MCF‐7, HepG2, and A549, were incubated in trypan blue (Invitrogen Inc., USA) for 5 min. After three PBS washes, the cells were examined under a microscope. Trypan blue‐stained cells were regarded as dead (Hoem et al. 2024).

### 2.19 Statistical Analysis

Data from all assays was statistically analyzed using Statistical Package for Social Sciences version 25 (SPSS, IBM, USA) and GraphPad Prism 10. Images were analyzed using Image J software.

## 3 Results

### 3.1 Proximate Analysis

The LC‐ Roots exhibited high moisture content (10.36% ± 0.05%), fat content (2.71% ± 0.01%), fiber content (18.40% ± 0.05%) and total carbohydrate content (37.27% ± 0.01%), whereas Ash (14.35% ± 0.01%) and protein content (26.91% ± 0.05%) were high in leaves (Table 1).

**TABLE 1:** Proximate composition of LC‐leaves and LC‐roots (%).

Parameters	LC‐leaves	LC‐ROOTS
Moisture	8.60 ± 0.01	10.36 ± 0.05
Ash	14.35 ± 0.01	13.90 ± 0.00
Nitrogen content	1.52 ± 0.01	0.96 ± 0.04
Protein content	26.91 ± 0.05	16.40 ± 0.03
Fat content	1.20 ± 0.00	2.71 ± 0.01
Fiber content	13.41 ± 0.05	18.40 ± 0.05
Total carbohydrates	34.01 ± 0.01	37.27 ± 0.01

### 3.2 Mineral Analysis

Mineral analysis was carried out to estimate the composition of micro‐nutrients in the LC‐Leaves and LC‐Roots (Table 2). The LC‐leaves were rich in iron, zinc, and sodium, whereas the concentration of magnesium and phosphorus was higher in the LC‐Roots.

**TABLE 2:** Minerals analysis of LC leaves and roots (mg/kg).

Minerals	Leaves	Roots
Zinc	34.26 ± 15.68	21.24 ± 6.23
Sodium	322.07 ± 18.23	254.21 ± 13.50
Phosphorus	1620.01 ± 19.04	2235.04 ± 7.23
Magnesium	1430.19 ± 16.50	2012.11 ± 10.13
Iron	9630.10 ± 43.24	5439.15 ± 26.85

### 3.3 Quantitative and Qualitative Phytochemical Analysis

The result of the quantitative phytochemical analysis revealed higher TPC (563.57 ± 1.44 mg GAE/g), TFC (284.59 ± 0.56 mg QE/g), and TAC (3.22% ± 0.57%) in the LC‐Root extract as compared to the LC‐Leaf extract (Table 3). Various phytocompounds, including coumarin, tannin, saponins, and triterpenoids, were confirmed through qualitative phytochemical analysis of the LC‐Leaf and LC‐Root extracts (Table 4).

**TABLE 3:** Phytochemical analysis, antioxidant activity, and free radical scavenging capacity of LC‐leaf and LC‐root extract.

	LC‐Leaf	LC‐Roots
Total phenol content (mg GAE/g)	433.19 ± 2.51	563.57 ± 1.44
Total flavonoid content (mg QE/g)	238.98 ± 1.26	284.59 ± 0.56
Total alkaloid content (%)	1.50 ± 0.69	3.22 ± 0.57
Total antioxidant activity (mg AAE/g)	1137.95 ± 1.39	1366.51 ± 2.98
Iron chelating ability (%)	17.27 ± 2.18	29.01 ± 2.40
FRAP (Abs. at 700 nm)	0.40 ± 0.02	0.69 ± 0.042
DPPH IC_50_ (μg/mL)	80.91 ± 0.54	178.921 ± 0.12
ABTS radical scavenging activity (% inhibition)	17.81 ± 0.39	71.47 ± 0.80

**TABLE 4:** Qualitative screening of LC‐leaf and LC‐root extract phytochemicals.

Active constituents	Positive results	Plant extracts	
		LC–leaf extract	LC–root extract
Coumarin	Cloudy	−	+
Tannins	White ppt	−	−
	Blue–black or green color	+	+
Triterpenoids	Red, purple or reddish	+	+
Saponins	Foam	+	+

Abbreviations: LC, *Lantana camara* ; ppt, precipitation.

### 3.4 Antioxidant Analysis

The antioxidant potential of LC‐Leaf and Root extracts was measured by iron chelating ability, FRAP, DPPH, and ABTS radical scavenging activities. The LC‐root extract not only exhibited a strong ABTS radical scavenging activity but also indicated an increase in iron chelating ability and ferric‐reducing antioxidant ability as compared to the Leaf extract, which only exhibited strong DPPH radical scavenging activity as indicated by the IC_50_ value.

### 3.5 Chemical Composition and Compound Analysis by GC–MS

Additionally, we identified the bioactive components included in the most effective solvent extract of *L. camara* using GC–MS analysis. Only the ethanolic root extract of LC was subjected to GC–MS profiling since it had a high concentration of phytochemicals and showed exceptional biological activity. The study discovered and isolated 10 recognized chemicals from various chemical classes (Table 5). The major compounds included lupeol (52.94%), hexadecenoic acid, methyl ester (0.746%), phytol (5.842%), phthalic acid, di(2‐propylpentyl) ester (0.861%), hexadecanoic acid (5.301%), spathulenol (1.797%), acetic acid, fluoro‐, ethyl ester (0.664%), 2,3‐dihydro‐2,5‐dihydroxy‐6‐methyl‐4H‐pyran‐4‐one (3.018%), germacrene‐D (1.993%), caryophyllene oxide (4.772%) and 9‐octadecatrienoic acid (Z)‐, methyl ester (0.103), Lup‐20(29)‐en‐3‐one (8.231%), 1‐Heneicosanol (3.75%), γ‐Sitosterol (6.98%) and neophytadiene (2.07%).

**TABLE 5:** Analysis of chemical composition of LC‐ethanolic root extract by GCMS.

Sr. No.	Compound name	Retention time	Percentage (%)
1	Lupeol	42.983	52.94
2	Hexadecanoic acid, methyl ester	38.464	0.746
3	Phytol	51.275	5.842
4	Phthalic acid, di (2‐propylpentyl) ester	73.480	0.861
5	Hexadecanoic acid	61.772	5.301
6	Spathulenol	35.857	1.797
7	Acetic acid, fluoro‐, ethyl ester	2.971	0.664
8	2,3‐Dihydro‐2,5‐dihydroxy‐6‐methyl‐4H‐pyran‐4‐one	27.834	3.018
9	Germacrene‐D	30.986	1.993
10	Caryophyllene oxide	39.099	4.772
11	9‐Octadecenoic acid (Z)‐, methyl ester	51.125	0.103
12	Lup‐20 (29)‐en‐3‐one	21.786	8.231
13	1‐Heneicosanol	19.392	3.75
14	γ‐Sitosterol	23.562	6.98
15	Neophytadiene	18.997	2.07

### 3.6 Morphology

**FIGURE 1:**
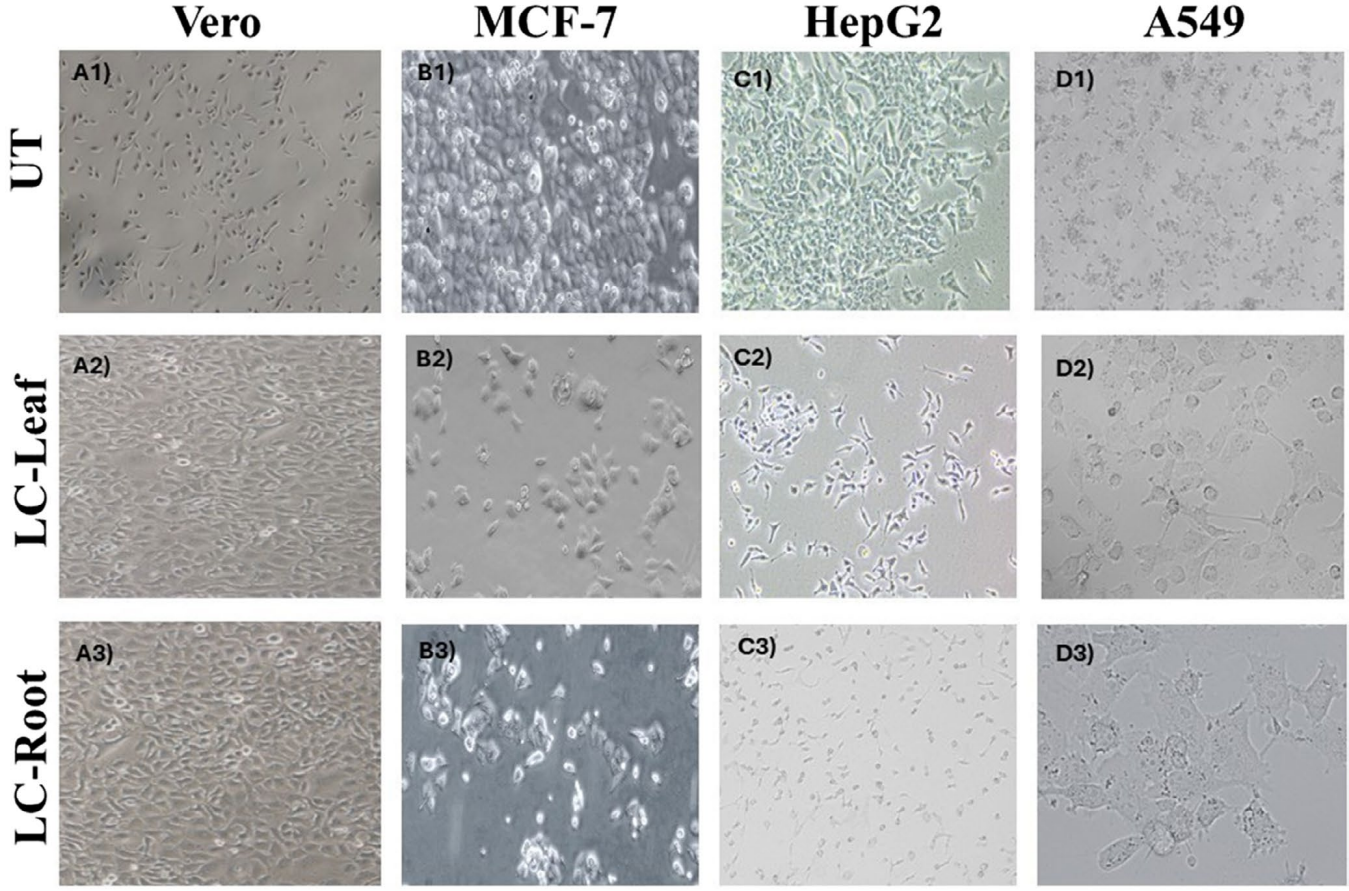
LC‐Leaf and LC‐Root extract effect on morphology of Vero (A1–A3), MCF‐7 (B1–B3), HepG2 (C1–C3), and A549 (D1–D3) cells. Images (10X) were analyzed by Image J software.

LC‐Leaf and LC‐Root extracts (50 μg/mL each) executed morphological changes to cancerous and normal cell lines: Images of the MCF‐7, HepG2, A549, and Vero cells were captured before and after the treatment. Before treatment, treatment each cell line showed optimal confluency from 70% to 80% and morphology was normal. Except for the normal cell line (Vero), each cancerous cell line showed shrinkage in cell morphology and compromised its adherent property (Figure 1).

### 3.7 Cytotoxic Analysis Through MTT Assay

**FIGURE 2.1:**
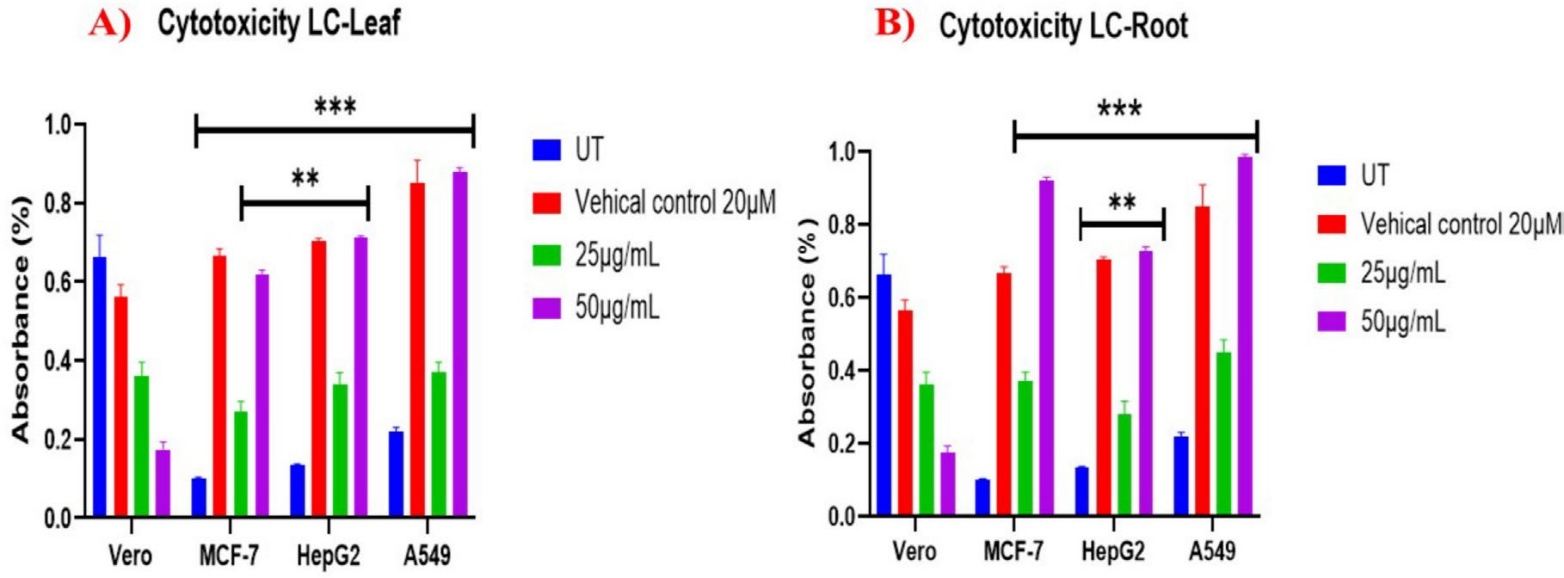
MTT cytotoxicity examination of (A) LC‐leaf extract (B) LC‐root extract. The data displays the mean ± standard deviation, with ** *p* < 0.001, and ***p < 0.0001 suggesting statistical significance compared to untreated and treated groups.

The cytotoxicity of LC was examined using 2 different concentrations of LC‐Leaf and LC‐Root extract (25 and 50 μg/mL) for 24 h. On application of LC‐Root and LC‐Leaf extract, MCF‐7, HepG2, and A549 showed significant cytotoxicity compared to the untreated group. Moreover, when LC leaf and root extracts were applied, the Vero cell line (non‐cancerous cells) showed reduced cytotoxicity. MTT assay exhibited higher cytotoxicity and anti‐proliferative activity at a 50 μg/mL concentration of both extracts when the cytotoxicity of 25 μg/mL LC‐Leaf extract was compared with 50 μg/mL on MCF‐7 (52.15%), a more significant *p*‐value (*p* < 0.0001) was obtained. At the same time, the highest significant cytotoxicity (*p* < 0.0001) was observed with 50 μg/mL on A549 cells (Figure 2.1A). Among LC‐root extract groups, the highest cytotoxicity was observed in A549 cells (*p* < 0.0001) and reduced cytotoxicity was noted in Vero cells (*p* = 0.05) (Figure 2.1B).

**FIGURE 2.2:**
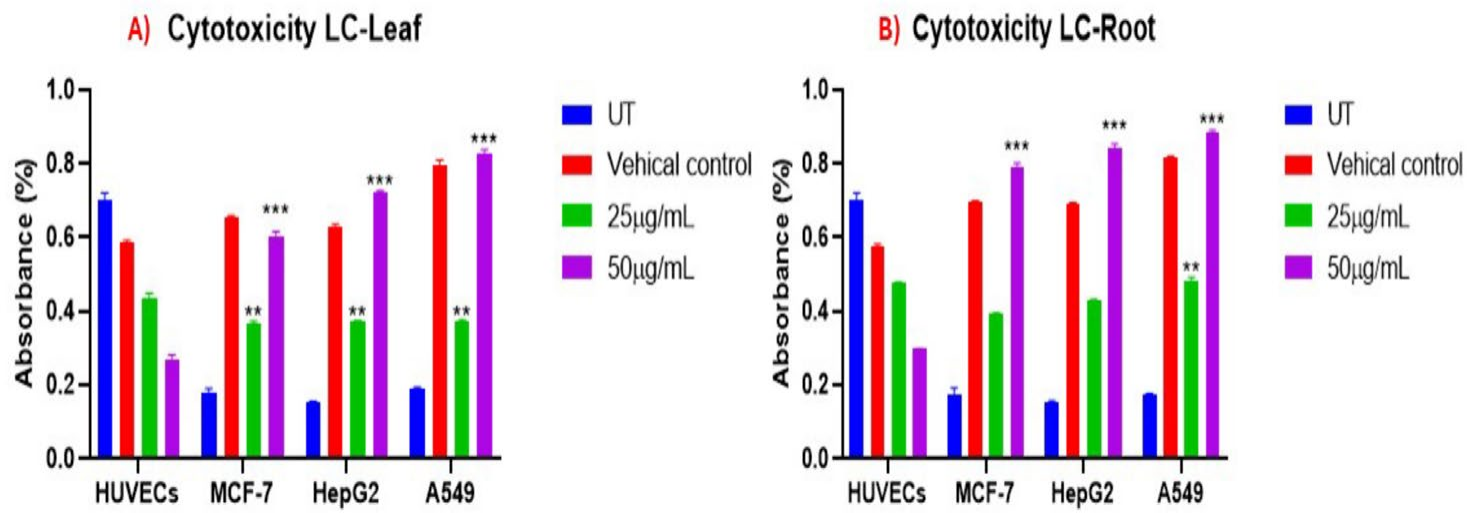
MTT cytotoxicity examination of (A) LC‐leaf extract (B) LC‐root extract with human umbilical vein endothelial cells (HUVECs). The data displays the mean ± standard deviation, with **p < 0.001, and ***p < 0.0001 suggesting statistical significance compared to untreated and treated groups.

Moreover, LC‐Leaf and LC‐Root extracts showed a lower cytotoxic impact (16.02%) on normal human umbilical vein endothelial cells (HUVECs) as compared to the cancer cells, confirming that LC extracts do not damage normal cells (Figure 2.2A,B).

### 3.8 Apoptotic Activity of LC‐Leaf and LC‐Root Extract

**FIGURE 3.1:**
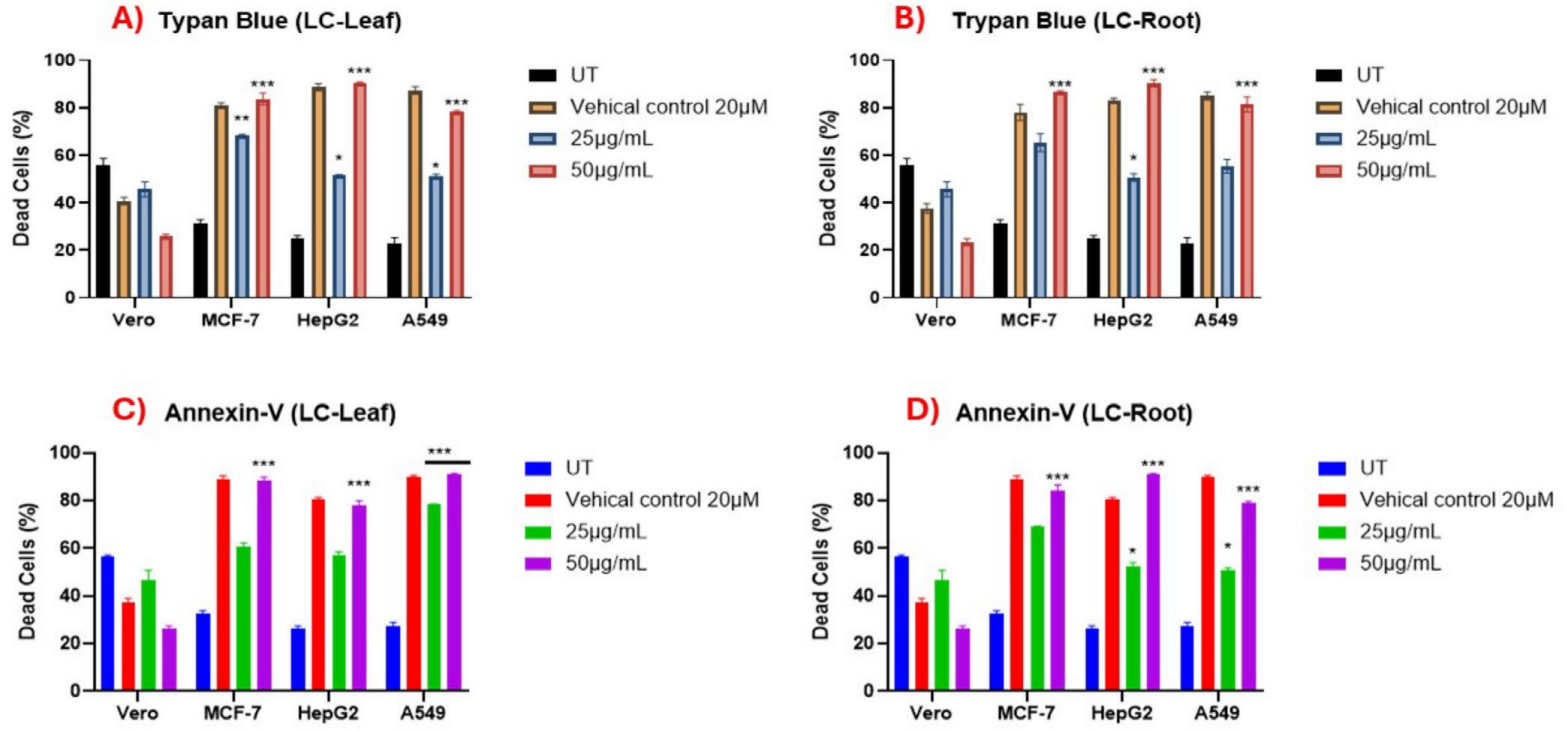
Cell death assay and expression of annexin V in cancerous and non‐cancerous cell lines with LC‐leaf and root extract. (A, B) Trypan blue assay for confirming the dead cells percentage for LC‐leaf and root extract. (C, D) Annexin‐V expression via ELISA in cancerous and Vero cell lines on treatment with LC‐leaf and root extract. All data was expressed via mean ± SD considering *p* < 0.05 as significant.

To determine the effect of LC‐Leaf and LC‐Root extracts, with two different concentrations; that is, 25 and 50 μg/mL, on the survival of Vero, MCF‐7, HepG2, and A549 cell lines, cells were assayed for the number of dead cell percentage by trypan blue and apoptotic antibody Annexin‐V (ELISA). The results of both the assays complemented each other; that is, a significantly large number of dead cells and increased apoptosis were observed in treated MCF‐7, HepG2, and A549 with LC‐Leaf extract (*p* < 0.0001) at 50 μg/mL. Compared to the untreated cell line Vero, fewer dead cells and more viable cells were seen, suggesting that LC‐Leaf and root extracts have no negative effects on normal cells (Figure 3.1A,C). More significant results were obtained in all treated cancerous cell lines on treatment with 50 μg/mL LC‐Root extract as compared to the untreated groups (*p* < 0.0001). The normal cell line Vero showed not much significance compared to the untreated group, confirming no harmful effect of LC‐Root extract on normal cells (Figure 3.1B,D).

**FIGURE 3.2:**
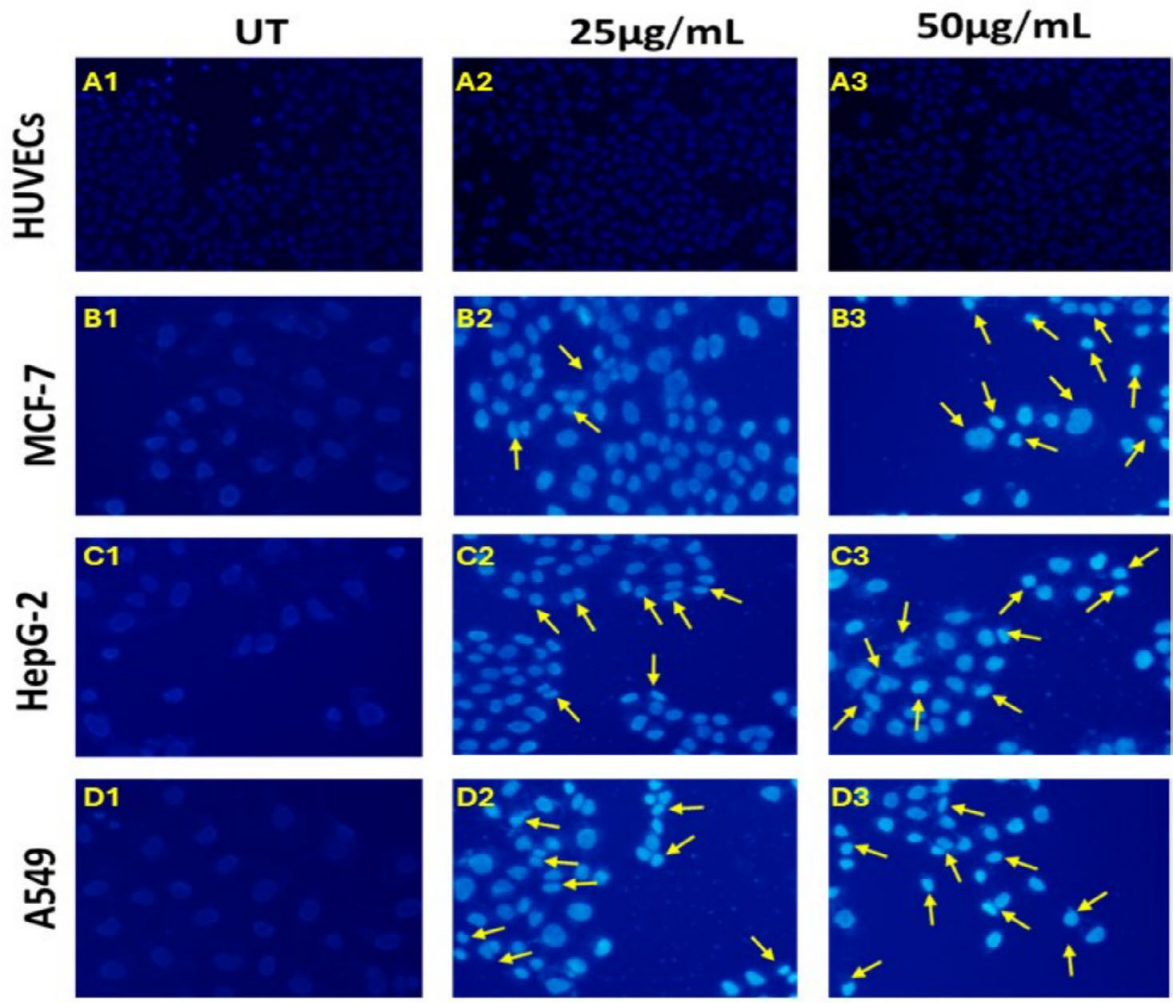
Hoechst 33342 staining to detect apoptosis in cancerous and non‐cancerous human cells with LC‐Leaf and root extract. (A1–A3) Normal human cells (HUVECs), (B1–B3) breast cancer cells (MCF‐7), (C1–C3) liver cancer cells (HepG2), and (D1–D3) lung cancer cells (A549). Apoptotic cells displayed more condensed nuclei (indicated by yellow arrow) in LC‐Root extract 50 μg/mL treated groups. Images were captured under fluorescence microscope and analyzed by Image J software.

Additionally, Hoechst 33342 staining revealed that when cancerous cell lines (MCF‐7, HepG2, and A549) were treated with 50 μg/mL of LC Root extract as opposed to 25 μg/mL of LC‐Root extract, the number of apoptotic cells increased; in contrast, normal HUVECs did not exhibit any discernible apoptotic cell shape. In treated carcinogenic cells, apoptotic cells had a spherical, compacted cell body, indicating that LC‐Root extract‐induced apoptosis of cancer cells may diminish cell viability but has no effect on normal cells (Figure 3.2).

### 3.9 Cell Viability Assessment by Crystal Violet Assay

**FIGURE 4:**
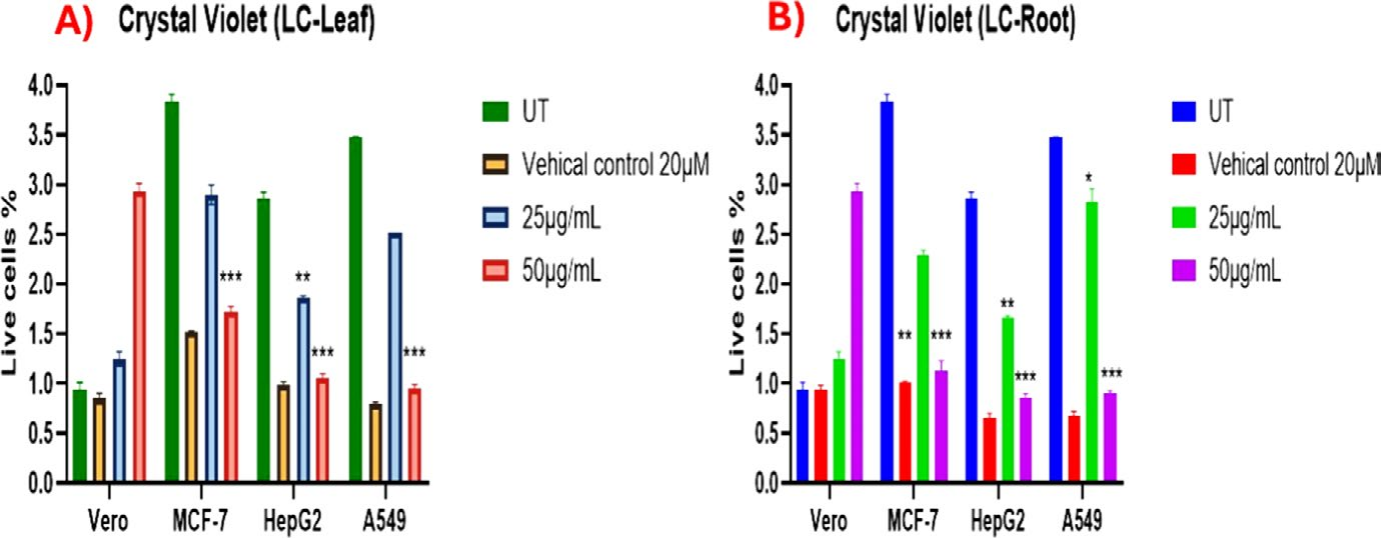
Crystal violet analysis of LC‐Leaf (A) and LC‐Root (B). The data displays the mean ± standard deviation, with *p < 0.05, **p < 0.001, and ***p < 0.0001 showing significance compared to untreated group.

Crystal violet assay was performed to analyze the viability of cancerous cell lines (MCF‐7, HepG2 and A549) and normal cell lines (Vero) after treatment with LC‐Leaf and Root extract. Both extracts showed significantly lower viable cell numbers in treated MCF‐7, HepG2, and A549 cell lines than in the untreated groups, whereas the normal cell line Vero showed high viability in treatment with LC‐Leaf and Root extract. Among the LC‐Leaf extract group, MCF‐7 showed slightly higher viability at the concentration of 50 μg/mL as compared to the treated LC‐Root extract group, which supports that LC‐Root extract has significant efficacy in reducing cancer at a 50 μg/mL concentration (Figure 4).

### 3.10 Assesment of Proliferation

**FIGURE 5:**
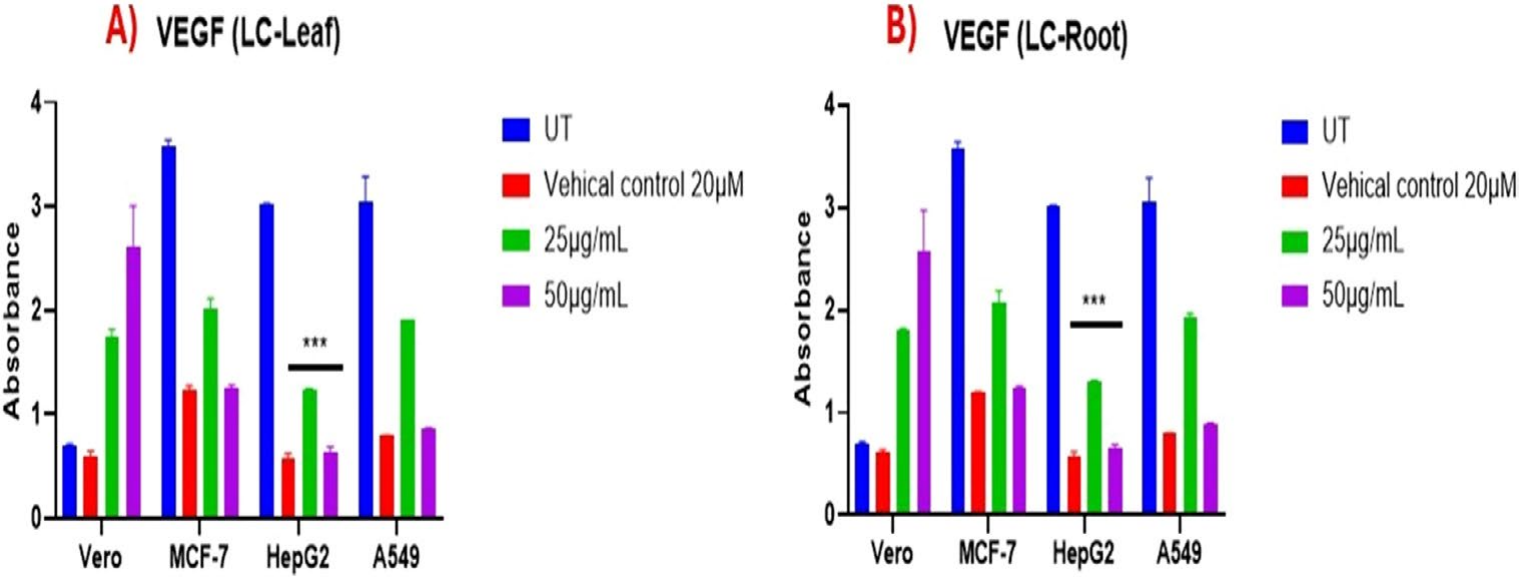
Expression analysis of VEGF in cancerous (MCF‐7, HepG2, and A549) and normal cell line Vero. The data displays the mean ± standard deviation, with **p < 0.001, and ***p < 0.0001 showing significance compared to untreated and treated groups.

To determine the effect of root and leaf extracts of LC on cell survival, Vero, MCF‐7, HepG2, and A549 cell lines were incubated with 25 and 50 μg/mL of both extracts. After incubation, ELISA assayed cells for VEGF (an angiogenic marker). Both extracts showed significantly lower proliferation (*p* < 0.0001) in treated cells; that is, MCF‐7, HepG2, and A549, as compared to the untreated and normal cell line group (V) (*p* > 0.05). The Vero cell line showed increased expression of VEGF on treatment with 25 μg/mL LC‐Leaf and LC‐Root extracts 25 and 50 μg/mL, which confirmed the proliferative effect of LC extracts on normal cells (Figure 5).

## 4 Discussion

*L. camara* (LC), commonly known as common lantana, is a flowering shrub with various uses and properties that can benefit humans. However, caution is advised due to its toxicity. LC is utilized in many traditional remedies and is widely distributed worldwide, possessing anti‐cancer medicinal properties mentioned in previous reports. Its high carbohydrate content suggests its potential as a valuable energy source, with carbohydrates, proteins, and lipids contributing to its overall energy, water, and ash (Etuh et al. 2021; Muindi et al. 2021; Ghosh et al. 2010). The present study found that the LC‐Root had elevated levels of moisture, lipid content, fiber content, and total carbohydrate content. Furthermore, the leaves exhibited elevated levels of ash and protein content. They are low in fat and contribute to hydration, energy, and digestive health. Their mineral richness and protein content make them a valuable food source, contributing to overall health and wellness. Incorporating such leaves into meals can enhance nutritional intake and support various health benefits. Roots contain high magnesium and phosphorus content Leaves and roots provide essential minerals for immune function, oxygen transport, bone health, and energy metabolism. Combining both parts in a diet maximizes nutrient intake and promotes overall well‐being (Madivoli et al. 2020). Analogous results from the current study show that leaves offer significant amounts of sodium, zinc, and iron. In comparison, roots are rich in phosphorus and magnesium.

Phenolic and flavonoid content in plants is linked to antioxidant activity, potentially promoting health benefits like anti‐diabetic, cancer, and inflammatory effects. Roots have higher flavonoids, alkaloids, antioxidant, and radical scavenging properties, making them a valuable addition to diets. Coumarin, tannins, and terpenoids are found in roots and leaves, with their distribution varying based on developmental stages and environmental conditions (Mansoori et al. 2020). Comparable results of the present study showed that roots contain high total phenolic content, total flavonoid content, total alkaloid content, total antioxidant activity, and ABTS radical scavenging activity in roots. The present study also showed that roots and leaves are good sources of coumarin, tannins, and terpenoids. A study has also reported that LC contains harmful compounds, such as lantadenes, which can cause serious health issues, including liver damage and gastrointestinal problems if ingested (Chen et al. 2024). It has not been recognized or used as a food source in any dietary practices. Its primary value lies in its medicinal properties and potential applications in areas like biofuels rather than as a source of nutrition. Consumption of this plant is strongly discouraged due to the risks associated with its toxicity (Pour et al. 2011). Therefore, in the current study, the effect of LC‐Leaf and LC‐Root extracts at 25 and 50 μg/mL did not show any toxic effect on the Vero cell line that was used as normal cells. This indicates that whole plants may have cytotoxic effects when taken in higher concentrations, whereas lower concentrations of extracts have no harmful effect on normal cells.

In GC–MS analysis of the phytoconstituent of *L. camara* ethanol root extract, hexadecenoic acid, methyl ester, has been shown in a recent study to have potent antibacterial and anticancer properties (Fayyad et al. 2022). Lupeol produces its anticancer effects by altering important signaling pathways such as PI3K/Akt, MAPK/ERK, and JAK/STAT and controlling the activity of proteins that promote cell cycle progression or apoptosis (Liu et al. 2021). Lupeol and Lup‐20 (29)‐en‐3‐one confirm a recent study suggesting that the extract possesses antioxidant and anti‐inflammatory qualities which enhance its anticancer efficacy (Zhang et al. 2022). The phytol found in the LC extract was found to have anticancer action by blocking the PI3K‐Akt signaling pathway (Yu et al. 2025). It is important to highlight that hexadecanoic acid and phthalic acid, di (2‐propylpentyl) ester, have potent anti‐inflammatory properties by blocking TNF‐α, a major contributor to inflammation (Heras Martinez et al. 2025). According to a recent study, spathulenol possesses anticancer properties and forms a strong hydrogen bond with the p53 protein (Satapathy et al. 2024).

LC extract showed remarkable anti‐cancer ability by inhibiting the proliferation of MCF‐7, HepG2, and A549 cells (cancerous cell lines). Still, it increased the proliferation in Vero cells (normal cell line) after 24 h of treatment with 50 μg/mL LC‐Leaf and Root extract. Among the two extracts, the LC‐Root extract showed better results than the LC‐Leaf extract.

Several human cancer cell types have been shown to respond positively to LC anti‐cancer effects, including the hepatoma cell line Huh7, cervical cancer cell line Hela, oral cancer cell line KP, prostate cancer cell line PC3, the brain cancer cell line A‐172, and the lung cancer cell line A549 (Fayyad et al. 2022; Liu et al. 2021). In the current study, similar effects were observed for LC‐Root extract at 50 μg/mL, which induced apoptosis, and expression of Annexin‐V and Hoechst 33342 stain was found to be increased in cancerous cell lines as compared to the untreated groups and normal cell lines (Vero and HUVECs).

LC is widely used worldwide to cure many conditions, including fever, rheumatism, chicken pox, ulcers, and asthma (Etuh et al. 2021; Zhang et al. 2022). Triterpenoids, one of *L. camara* 's bioactive ingredients, have been shown to have cytotoxic effects on a variety of malignancies, including breast cancer, at the cellular level (Yu et al. 2025; Heras Martinez et al. 2025).

This investigation found that LC‐Leaf and Root extract altered the morphology of all three cancer cell lines: MCF‐7, HepG2, and A549. However, Vero cells exhibited no morphological or confluency changes. The extract caused a discernible change in the morphologies of the cells, which was suggestive of cytotoxic stimulation. The cytotoxic effect was shown to be mediated by a decrease in cell viability, according to experimental results from cell viability experiments. Under normal physiological settings, the delicate balance between cellular proliferation and progression through the cell cycle is maintained. It was discovered that the extract's ability to prevent malignant cells from proliferating is related to its ability to induce cell death.

Numerous plant‐based chemotherapeutic medications, including vincristine, sulforaphane, and paclitaxel, have been efficacious in treating cancer (Satapathy et al. 2024). Curcumin, genistein, resveratrol, and lycopene are natural compounds that have demonstrated cytotoxic and anti‐migratory actions on triple‐negative breast cancer (Tufail et al. 2024; Bisi‐Johnson et al. 2011). The results of this study demonstrated that LC‐leaf and LC‐Root extract can inhibit the motility of malignant cells in addition to inducing cellular death, hence counteracting the aggressive nature of these cells. The primary reason for the anti‐proliferative impact of natural plant products is the existence of bioactive substances such as phenols, terpenoids, and flavonoids (Hoang et al. 2024; Kacholi 2024; Kaur et al. 2008). Terpenoids, flavonoids, phenols, and steroids were discovered to be present in the ethanolic extract of LC leaves employed in this investigation based on early chemical tests. Though the cytotoxic activity of the LC extract used in this study showed higher activity at 50 μg/mL, similarly to what was reported by some studies that compared the LC extract with purified natural products like doxorubicin (Shamsee et al. 2019) and paclitaxel (Mazumder et al. 2022), it can be conjectured that the LC‐Root and LC‐Leaf could be a pretentious chemotherapeutic comparable to those presently being utilized in cancer treatment.

## 5 Conclusion

A current study revealed the phytoconstituents of LC‐Leaf and LC‐Root ethanolic extract have antioxidant properties. Both extracts induced cytotoxic effects on breast cancer, liver cancer, and lung cancer cell lines, while normal cell lines (HUVECs and Vero) were unaffected by the extracts used in 25 and 50 μg/mL concentrations. Among LC‐Leaf and LC‐Root extracts, better anti‐cancerous effects led to apoptosis‐mediated cellular death assessed by Annexin‐V and Hoechst 33342 staining. Proliferation and cytotoxicity were assessed by VEGF and MTT, which showed lower proliferation in post‐treatment cancerous cells and decreased cytotoxicity in normal cell lines used as control. Future studies should concentrate on in vivo validation to assess systemic toxicity, pharmacokinetics, and therapeutic efficacy using suitable animal models.

## Author Contributions

**Somia Shehzadi:** writing – original draft (equal). **Sana Noreen:** data curation (equal). **Hassan Imran:** formal analysis (equal). **Ali Ikram:** supervision (equal). **Muhammad Tayyab Arshad:** writing – review and editing (equal). **Kodjo Théodore Gnedeka:** validation (equal).

## Disclosure

The authors have nothing to report.

## Consent

This study did not involve humans.

## Conflicts of Interest

The authors declare no conflicts of interest.

## Data Availability

The data supporting this study's findings are available from the corresponding author upon reasonable request.
